# Effect of the Growth Conditions on Organic Crystals with Rare Earth Ions and 1,10-Phenanthroline

**DOI:** 10.1021/acsomega.4c00526

**Published:** 2024-04-23

**Authors:** Ashleigh K. Wilson, John Munga, Tori Furlow, Violet Macauley, Jordan Graham, Asia Jones, Chantel Johnson, Natalia Noginova

**Affiliations:** Center for Materials Research, Norfolk State University, Norfolk, Virginia 23504, United States

## Abstract

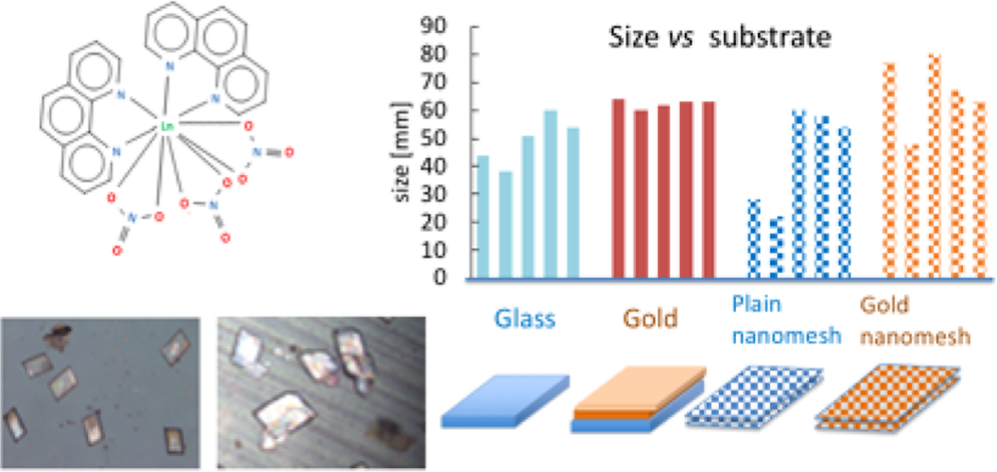

Using a simple solution growth technique, we grow crystals with phenanthroline as a ligand and various rare earth ions: thulium (Tm), ytterbium (Yb), gadolinium (Gd), lanthanum (La), neodymium (Nd), europium (Eu), and erbium (Er). We then selected the composition that forms thin plates with well-defined shapes, Er(NO_3_)Phen_2_, and explored the effects of various conditions on crystal formation and growth, including temperature regime, light illumination, and substrates where the crystals are formed and grown. The composition and local environment strongly affect the size and shape of microcrystals and substrate coverage. The use of gold substrates significantly enhances the crystal growing process. Elevated temperatures negatively affect the crystal growth.

## 1 Introduction

Luminescent materials with rare earth ions present interest for various applications ranging from light sources to biomedical probes.^1,2^ Organic materials with lanthanides are convenient systems for many nanophotonic applications. They can be easily synthesized and provide an opportunity for thin film fabrication and nanofabrication, including the deposition of the luminescent material in a particular location in resonance cavities or nanostructures.^3^ The optical properties of rare earth ions in such systems can be optimized by choosing the ligand, which may allow one to significantly expand the excitation range, since organic ligands have excellent light absorption abilities and can transfer energy to lanthanide ions. In addition, the ligand can enhance the emission due to the so-called “antenna effect”.^4^ As known, f-orbital to f-orbital electron transitions of lanthanide ions are forbidden by the parity rule. Thus, their fluorescent intensities and quantum efficiencies are commonly low, as observed, for example, in lanthanide(III) nitrates. The “antenna effect” associated with the presence of certain organic ligands allows for the lanthanides to partially disobey the parity rule, resulting in increasing the luminescence efficiency of the compound.

Organic systems with rare earth ions have multiple applications. The electroluminescence and mechanoluminescence of lanthanide(III) complexes via excitation of the ligand and energy transfer have been studied for the development of display devices. The f-orbital to f-orbital emission of Tb^3+^, Eu^3+^, and Sm^3+^ plays a vital role in the design of monochromatic green, red, and deep-red luminescent materials for displays, lighting, and sensing devices.^6^ The emission of Yb^3+^, Nd^3+^, and Er^3+^ in the near-infrared region presents interest for bioimaging and security applications.^7^ Recently, materials with rare earth ions have received much attention due to the presence of magnetic dipole (MD) transitions in their absorption and emission spectra.^8−13^ Materials with MD transitions are of high interest for optical magnetism studies and provide the possibility to probe the magnetic interactions of light and matter. Materials with Eu^3+^ ions are the most popular systems for such studies since Eu^3+^ ions show a distinct and relatively strong MD transition ^5^*D*_0_ → ^7^*F*_1_ at 590 nm.^8^ Noticeable MD transitions are common for other ions: Gd^3+^ ions have a ^8^S_7/2_ → ^6^D_9/2_ MD transition in the UV region at 253 nm, while some MD transitions contribute to infrared luminescence like that of Er^3+^, which has a ^4^*I*_15/2_ → ^4^*I*_13/2_ transition simulated to be around 1528 nm, and Tb^3+^ possessing a ^7^F_6_ → ^7^F_5_ transition calculated to be a little over 5000 nm.^13,14^ Eu(NO_3_)_3_(Bpy)_2_ as a material has been extensively used in optical magnetism studies.^8,9,12,15^ It was also shown that it is possible to produce crystals with various ions and 2,2-bipyridine (Bpy) and 1,10-phenanthroline (Phen).^16−19^ The photoluminescent properties of these compounds depend on the crystal structure and the Ln/ligand reactant ratio.^17−19^ This occurs when lanthanide nitrates are attached to a ligand and become polarized, resulting in an increase in absorption efficiency, modifications in energy gaps between the HOMO and LUMO, and more efficient excitation energy transfer from the ligand to the emitting ion.^18^ Hussain et al. studied lanthanide nitrates grown with Bpy as the antenna ligand and explored their potential for use in optical studies. It was found that crystals with different rare earth ions can be excited at UV and have efficient energy transfer from the ligand to the rare earth ion.

**Figure 1:**
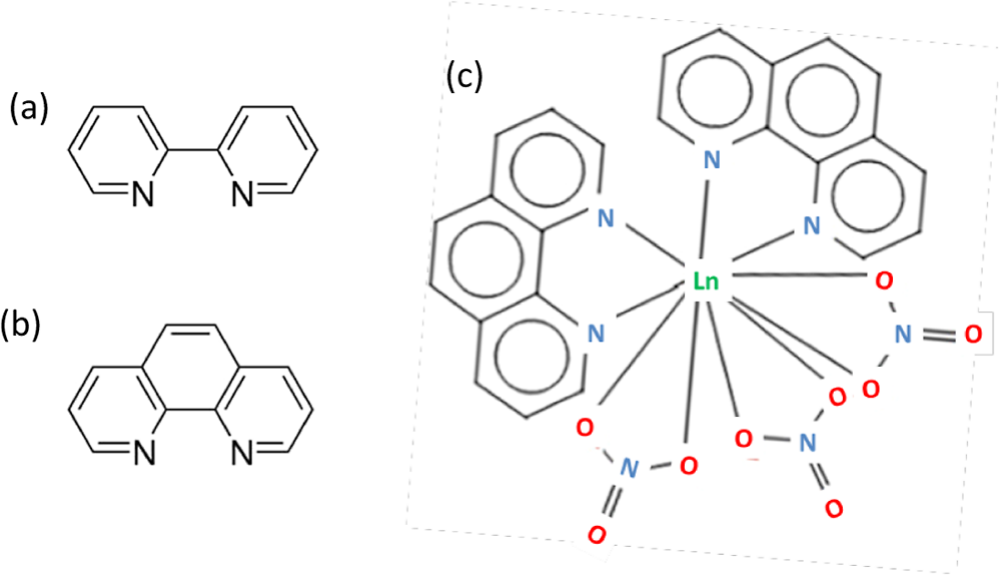
Structures of (a) 2,2-bipyridine, (b) 1,10-phenanthroline, and (c) Ln(NO_3_)_3_(Phen)_2_.

Unlike bipyridine, phenanthroline is a rigid ligand in which the pyridyl groups are linked together through an aromatic system,^20^ see Figure 1. 2,2-Bipyridine has been found to be less basic than that of Phen, with the pH value of Bpy being 4.30 and the pH of Phen being 4.86. 1,10-Phenanthroline is an organic, bidentate ligand that entails several appealing structural and chemical properties such as rigidity, planarity, aromaticity, basicity, and chelating capability that make it a versatile starting material for supramolecular chemistry. It absorbs in the ultraviolet range and has a strong affinity for multiple lanthanide(III) ions, particularly Eu^3+^. The rigid aromatic phenyl and pyridine rings on Phen have the potential to generate π–π and C–H−π stacking interactions, which significantly impacts the crystal packing and influences the architecture of the resulting crystal structures.^20^ Crystals grown using rare earth complexes and Phen have been fabricated and studied in ref (5,17–19). In ref (5) crystals were fabricated using the phenanthroline complex of erbium nitrate and found to have two crystalline modifications with the same chemical composition.

In this work, we grow crystals with various rare earth ions using phenanthroline as a ligand, including systems with well-defined MD transitions, which can be used in optical magnetism studies. However, the main goal of this work is to explore the effects of various conditions on crystal formation and growth, including the use of different rare earth ions and environmental conditions. Recent developments in the field of metamaterials demonstrate the ability to control a variety of physical and chemical processes via local optical environments.^21−28^ The most well-known effect is the acceleration of spontaneous emission rates in a plasmonic environment (the Purcell effect) via a modification in the photonic mode density.^21^ In addition, it is shown that processes such as energy transfer, charge transfer, and van der Waals forces can be strongly altered in close vicinity of plasmonic structures and metamaterials.^22,23^ In plasmonic structures, chemical and electrochemical reactions are found to be modified as well, due to a variety of mechanisms, including the effects of hot electrons, the charging of nanostructured metals, and the modification of work functions.^23−27^ Optical illumination and local heating can be a factor as well.^28^

As the literature suggests, organic crystals with rare earth are very sensitive to growth conditions. In ref (16) typical sizes of crystals strongly varied depending on the ambient temperature during the crystal growth process. The shape of the crystals can be affected by the local environment as well.^5^ In this work, we use the solution growth process as a playground for the exploration of a range of factors, including the plasmonic environment, light illumination, and temperature, on the formation of microcrystals and crystal growth. The goals of the work are (i) to study possible effects of the environment, in particular, the vicinity of plasmonic metals and light, on physical and chemical processes involved in the formation and growth of microcrystals, and (ii) to discover an optimal procedure for growing crystals that are useful in various plasmonic studies since they are stable, suitable for thin film deposition, and have a relatively high yield of luminescence. Below, the methodology and then the results are presented.

## 2 Experimental Methods

### 2.1 Growth of Organic Crystals with Various Rare Earth Ions and Phenanthroline

In this experiment, organic crystals Ln(NO_3_)_3_(Phen)_2_ are grown using a solution growing technique and various rare earth ions. The chemicals with rare earth ions (Table 1) and phenanthroline are obtained from Sigma-Aldrich. The process of solution growth described below is replicated for each rare earth ion in order to produce microcrystals with phenanthroline and different ions: thulium (Tm), ytterbium (Yb), gadolinium (Gd), lanthanum (La), neodymium (Nd), europium (Eu), and erbium (Er).

**Table 1:** Compounds and Molar Masses

compounds	molar mass (g/mol)
phenanthroline (C_12_H_8_N_2_)	180.22
Eu(NO_3_)_3_·5H_2_O	428.09
Er(NO_3_)_3_·5H_2_O	443.39
Nd(NO_3_)_3_·6H_2_O	438.39
Gd(NO_3_)_3_·6H_2_O	451.40
Tm(NO_3_)_3_·5H_2_O	445.06
La(NO_3_)_3_·6H_2_O	433.06
Yb(NO_3_)_3_·5H_2_O	449.17

First, the molar mass of each compound is calculated. The ethanol solutions with 0.1 M for a rare earth compound and 0.2 M of phenanthroline are prepared in separate vials and sonicated for 30 min. Then, the two vials were mixed in one beaker and sonicated for an additional 15 min to achieve a homogeneous solution. The top of the beaker is covered with parafilm, leaving a small opening for solvent evaporation. The beakers filled with the solutions prepared for each rare earth material are left in the fume hood at room temperature for several days to allow for the crystals’ growth. The crystals formed can be seen as a white residue on the bottom and walls of the beakers. The shape and typical sizes of the crystals are analyzed with an optical microscope. For spectroscopic analysis, the crystals are placed between two thin quartz substrates, and the absorption spectra of each of the samples are recorded using a standard PerkinElmer Lambda 900 UV/vis/NIR spectrometer. Excitation and emission spectra in the visible range are recorded with the fluorometer, HORIBA Spectrofluorometer Fluoromax-3/Fluorolog-3.

### 2.2 Study of the Effects of Various Conditions on Microcrystal Formation and Growth

For this experiment, one type of crystal, Er(NO_3_)_3_Phen_2_ (which tends to grow in the shape of thin plates), is used to grow the crystals in various conditions.

**Figure 2:**
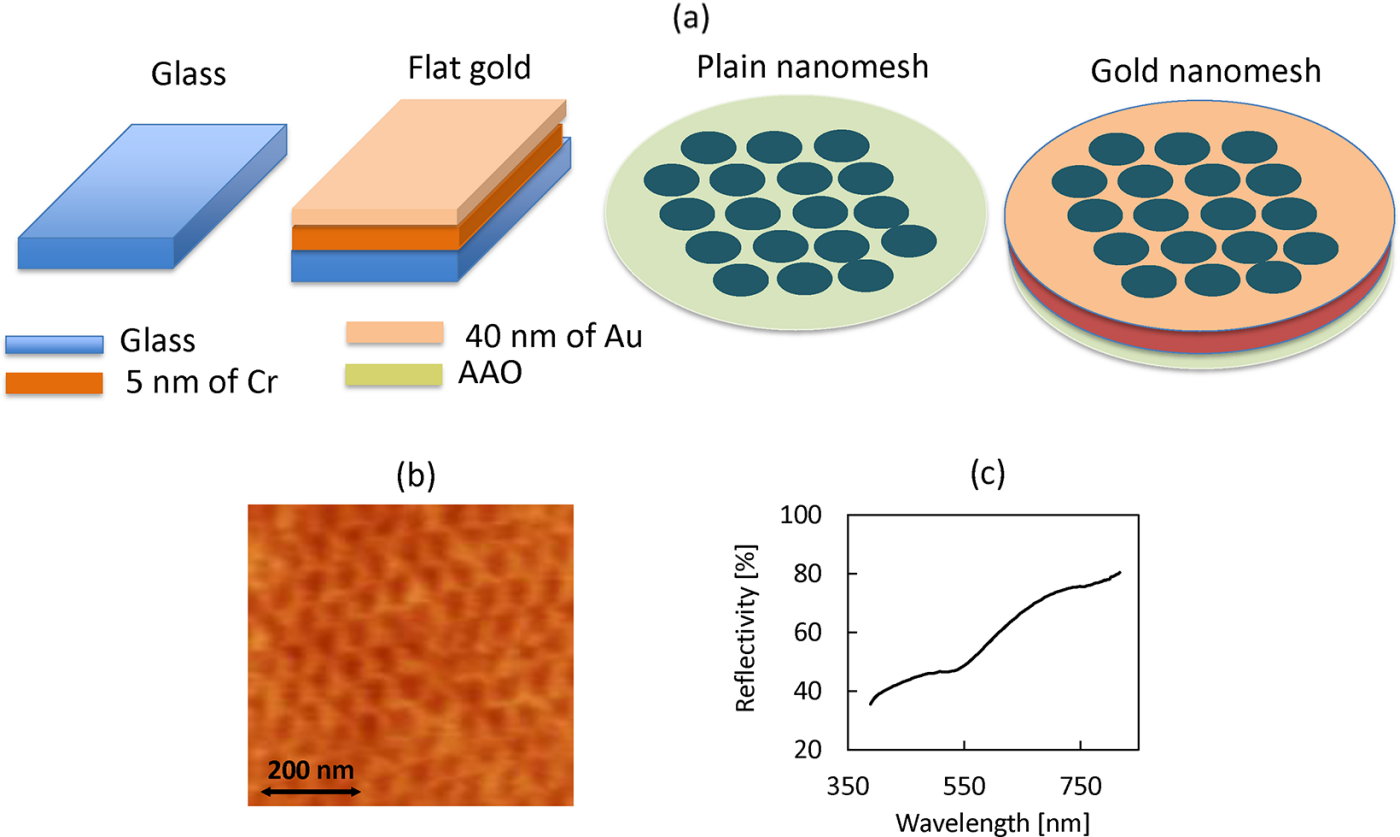
(a) Schematics of the structures; (b) atomic force microscopy; and (c) reflectivity of the gold nanomesh.

To explore different regimes, the following varying parameters are used:

1. Temperature: ∼ 22 °C (ambient), ∼ 3 °C (cold), and ∼60 °C (elevated).
2. Light illumination: ambient (room light), dark, and additional laser light.
3. Substrate: glass, flat gold, nanostructured alumina (plain nanomesh), and gold nanomesh, see Figure 2.

The low temperatures are achieved using a commercial refrigerator, and a hot plate was used for elevated temperatures. The laser illumination is provided by a diode laser emitting at 632 nm with a power of 114 mW; the size of the spot is about 1.2 mm. The substrates, illustrated in Figure 2a, are prepared by the following methods. The glass substrates (microscope slides) are cleaned and dried. The plain nanomesh makes use of porous anodic alumina oxide (AAO) membranes with a nominally 40 nm pore diameter. The glass slides and AAO membranes (purchased from InRedox) were nominally 40 nm in pore diameter. The membranes have the shape of a disk with a 13 mm diameter and 50 μm thickness. The glass slides and AAO membranes are precleaned and kept in a clean specimen container before further use. The flat gold substrates are obtained with the microscope slides coated with an adhesion layer of 5 nm thick Cr and 40 nm thick Au, using the thermal evaporation method. The layers of Cr and Au are deposited on the AAO membranes in the same deposition step. The thickness of the layers is confirmed with the profilometer measurements on the flat systems. The deposition of the Au onto the membranes produces highly nanostructured metasurfaces featuring plasmonic behavior with resonances in visible and infrared. Note that similar substrates have been used in the electrochemical experiments,^23^ where they were found to significantly affect the electrochemical reaction rates.

The substrates (glass, gold, plain nanomesh, and gold nanomesh) were placed on the bottom of four separate containers (round beakers with a diameter of 4 cm) to test the effect of various growth conditions and substrates in isolated environments. As initial solutions, a 0.0125 M solution Er(NO_3_)_3_·6H_2_O and a 0.025 M solution of Phen were used. In comparison to the first experiment, they are more diluted to obtain dispersed crystals. After mixing the two solutions, the resulting solution is poured into the containers with different substrates on the bottom and quickly placed under the various conditions (different temperatures and light illumination). The substrates with the crystals formed on them are carefully extracted and analyzed 30 min after this moment.

## 3 Results

### 3.1 Effect of Composition (Rare Earth Ion on Crystal Properties)

**Figure 3:**
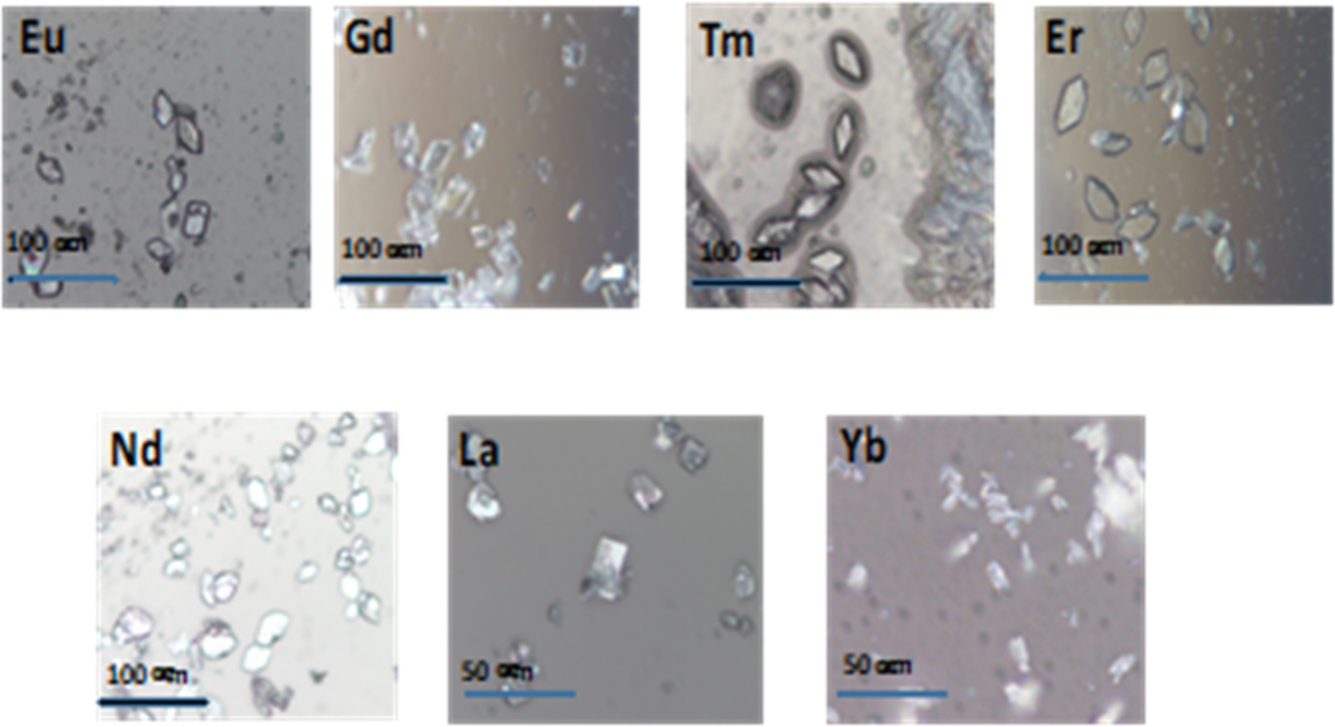
Microscope pictures of Ln(NO_3_)_3_(Phen)_2_, here Ln is Eu, Gd, Tm, Er, Nd, La, and Yb as indicated.

The obtained crystals have various shapes and sizes (Figure 3), depending on which rare earth is used. Crystals with Er and Nd look like flat flakes; crystals with Tm have well-defined parallelogram shapes; crystals with La and Yb are the smallest ones (5–10 μm) with irregular shapes; the other crystals grow up to ∼20–60 μm in length. For an estimation of the size of the microcrystals, of about 40–60 individual crystals have been measured using a ruler on microscope images where the crystals can be clearly seen, being considerate of the magnifications.

**Figure 4:**
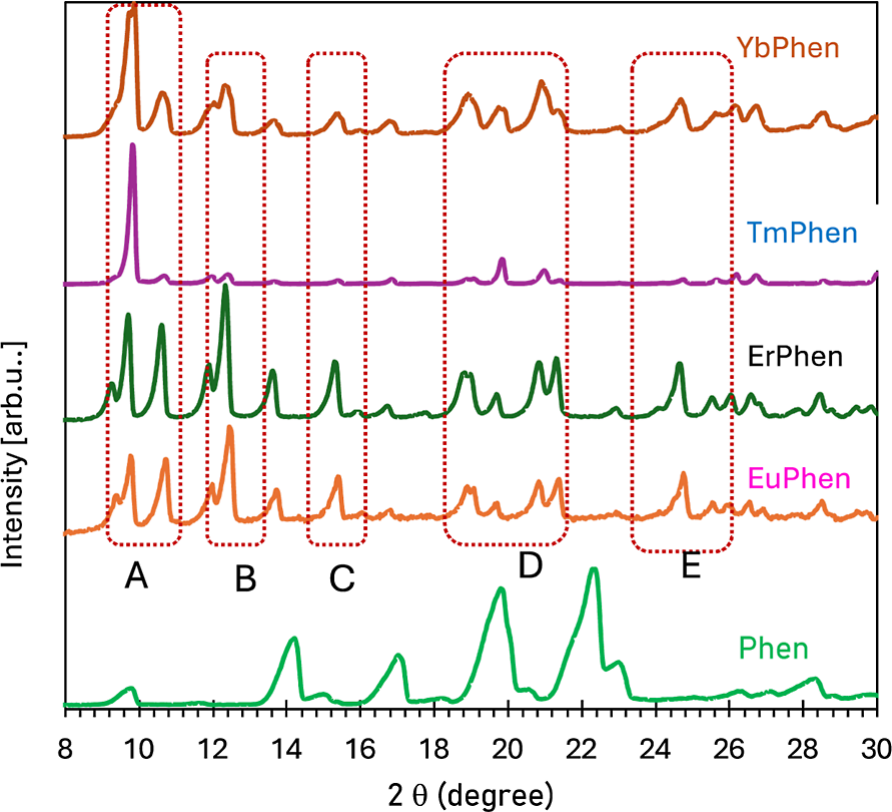
X-ray diffraction in YbPhen, ErPhen, EuPhen, and TmPhen and phenanthroline.

The results of X-ray diffraction measurements of YbPhen, ErPhen, EuPhen, and TmPhen are shown in Figure 4. The crystals demonstrate patterns similar to each other and different from those in phenanthroline. The crystals show main peaks around 10 (A), 12 (B), 15.3 (C), 20 (D), and 26° (E). They well correspond to the pattern observed in Ce(NO_3_)_3_(Phen)_2_ synthesized and analyzed in ref (24), indicating that synthesized crystals have a similar crystal structure.

**Figure 5:**
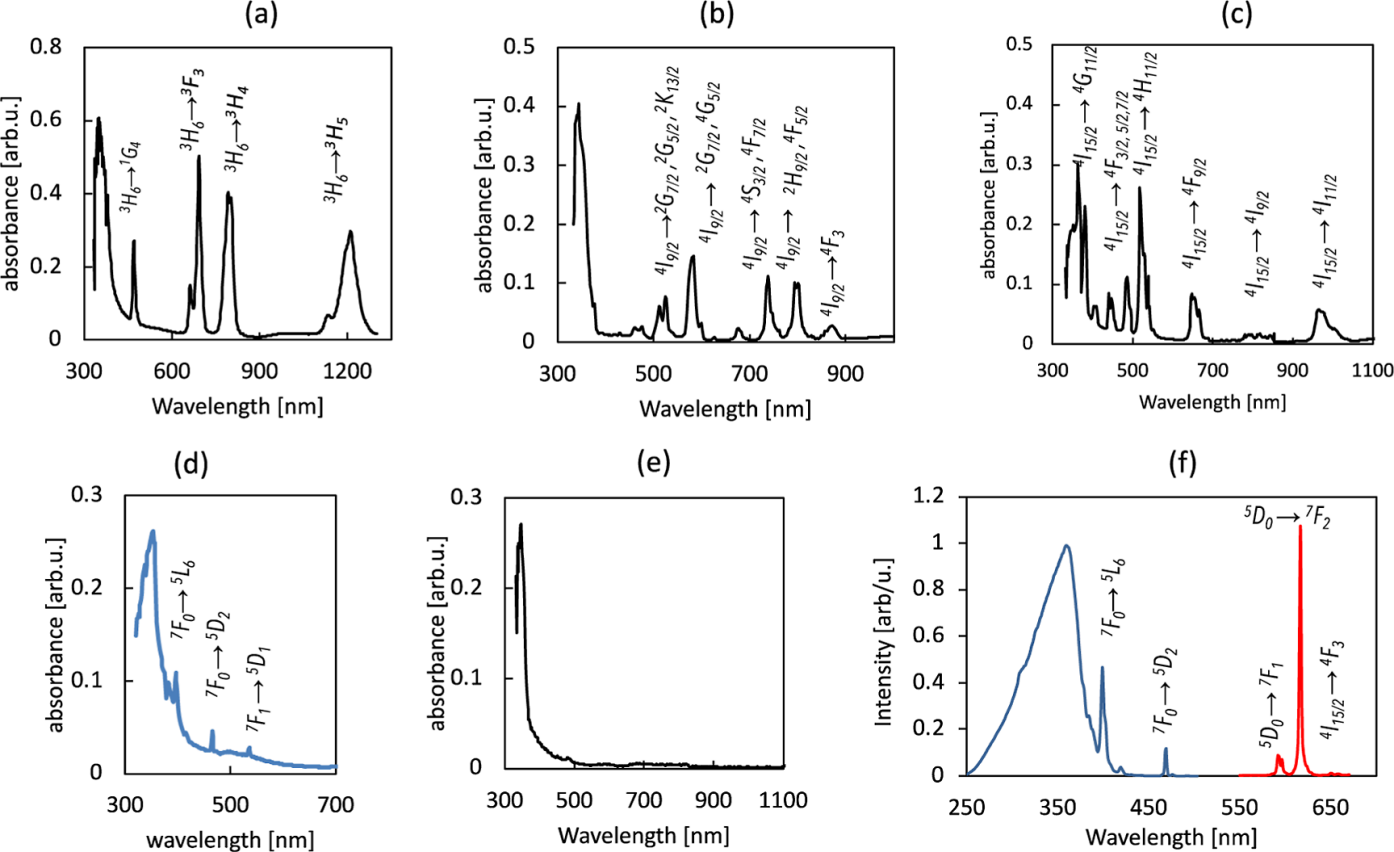
(a–e) Absorption spectra of (a) Tm(NO_3_)Phen_2_, (b) Nd(NO_3_)Phen_2_, (c) Er(NO_3_)Phen_2_, (d) Er(NO_3_)Phen_2_, and (e) phenanthroline. (f) Excitation (blue) and emission (red) spectra of Eu(NO_3_)Phen_2_.

The spectroscopic measurements confirm the presence of three-valent rare earth ions and phenanthroline in the crystals. Figure 5a–d depicts the absorption spectra of the crystals with Nd, Tm, Er, and Eu. The presence of the characteristic absorption peaks of ions in the crystals is seen, corresponding to the excitations from the ground state ^4^I_9/2_ for Nd, ^3^H_6_ for Tm, ^4^I_15/2_ for Er, and ^7^F_0,1_ for Eu. Crystals of Yb(NO_3_)Phen_2_ have absorption around 970 nm associated with ^2^F_7/2_ → ^2^F_5/2_ transmission; Gd(NO_3_)Phen_2_ and La(NO_3_)Phen_2_ do not show any absorption lines in the range studied except of the strong absorption peak around 340–350 nm. This absorption peak is observed at all the materials and associated with the ligand absorption (compare with the absorption spectrum of the phenanthroline, Figure 5e). The presence of the Phen ligand in the composition of these crystals is expected to boost the emission, via excitation energy absorption by the ligand, energy transfer to the rare earth ion, and an efficient antenna-stimulated luminescence.^4^ In fact, Eu(NO_3_)_3_(Phen)_2_ is bright red under UV light, showing strong emission in the visible. The excitation and emission spectra of Eu(NO_3_)_3_(Phen)_2_ are shown in Figure 5f. As can be seen, the excitation peak corresponds to the absorption of the ligand. As expected, the strongest emission peak at 613 nm corresponds to the electrical dipole transition ^5^*D*_0_ → ^7^*F*_2_, and the line at ∼590 nm is the magnetic dipole transition ^5^*D*_0_ → ^7^*F*_1_. Thus, in Eu(NO_3_)_3_(Phen)_2_, the ligand provides a possibility for efficient excitation with UV light. Emission and excitation studies of other produced materials are in progress and will be published elsewhere.

### 3.2 Effect of Environmental Conditions

Using an optical microscope, the Er(NO_3_)_3_(Phen)_2_ microcrystals produced with the diluted solution on the substrates are inspected and analyzed. Crystals strongly differ in shape, size, and coverage of the substrate depending on the substrate and environmental conditions.

**Figure 6:**
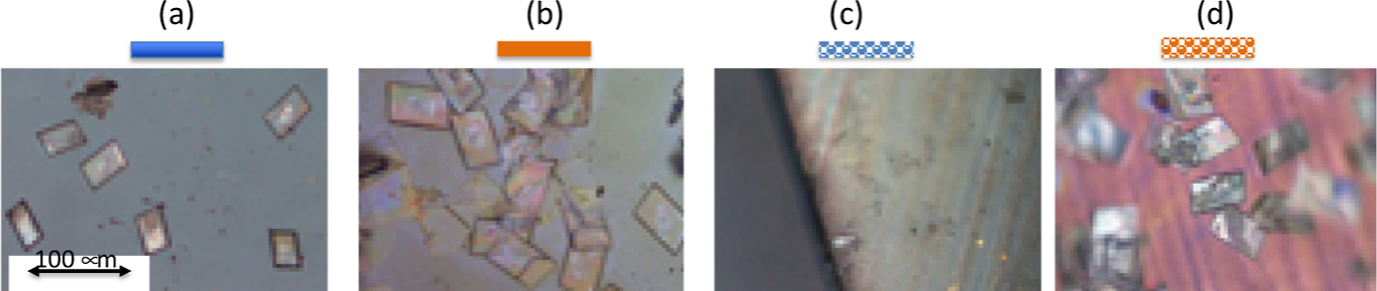
Crystals grown under standard conditions using (a) glass, (b) flat gold, (c) plain nanomesh, and (d) gold nanomesh.

Figure 6 shows images of the crystals grown at standard conditions (room temperature and illumination) on glass, gold, plain nanomesh, and gold nanomesh substrates. The crystals observed on the glass are well-defined and are almost of the same size; see Figure 6a. Gold seems to aid in the production of crystals, as they are in abundance and lead to an increase in their size, as shown in Figure 6b. The plain nanomesh, Figure 6c, barely produces any crystals; they are far away from each other and are smaller when compared to any of the other cases. The crystals on the gold nanomesh (Figure 6d) are similar to those on the flat gold but slightly smaller in size.

**Figure 7:**
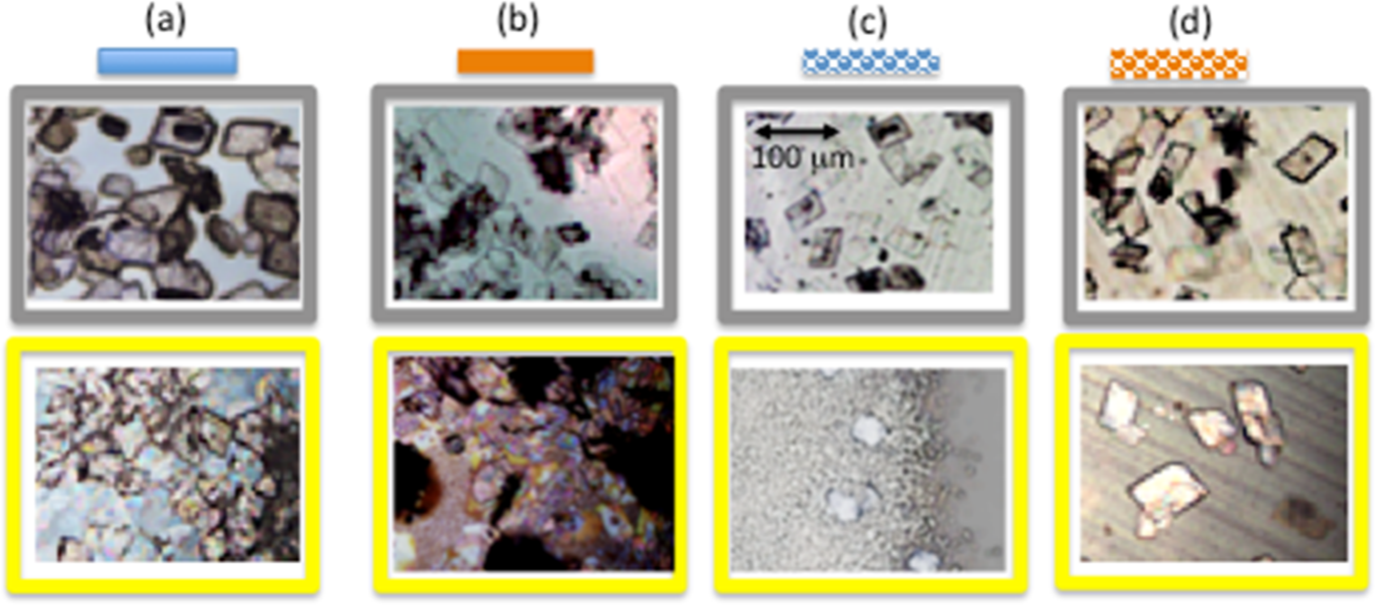
Typical crystals grown in dark conditions (top row) and at room light with additional laser light illumination (bottom row) using (a) glass, (b) flat gold, (c) plain nanomesh, and (d) gold nanomesh substrates. Scale bar (same for all images) is shown in the figure (c) top row.

In the work, light’s possible effect on microcrystal formation and growth is also explored. Typical images of the crystals grown in dark conditions and with additional laser light focused on a central section are shown in Figure 7. The microcrystals grown in the dark are comparable in size to those grown under standard conditions, and additional light illumination is required for most of the cases. The crystals are abundant on the gold surfaces, and less abundant on the plain nanomesh, in similarity with the results, as shown in Figure 6.

**Figure 8:**
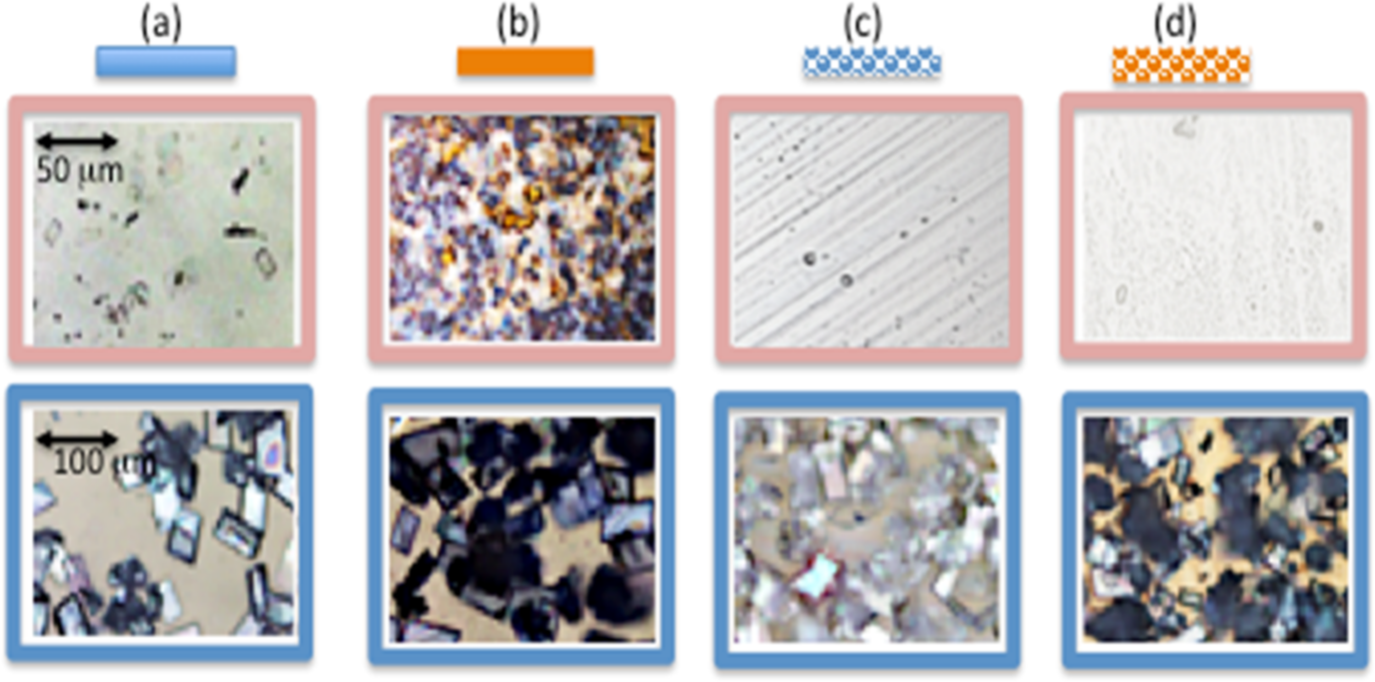
Typical crystals grown at elevated temperature (top row) and in freezer with (bottom row) using (a) glass, (b) flat gold, (c) plain nanomesh, and (d) gold nanomesh substrates. Scale bars for the top row and bottom row are different and shown in (a) top and (b) bottom pictures correspondingly.

The crystals grown at elevated temperatures (Figure 8, top row) have more of a spherical or irregular shape rather than rhomboidal. This is in accordance with the results of ref (5) where different shapes are observed for room temperature and elevated temperature. The sizes of the crystals for all substrates decreased greatly. Less number of crystals are formed on the glass and nanomesh substrates. However, there is still good coverage on plain gold. On the contrary, at low temperatures (Figure 8, bottom row), the crystals are readily formed at all substrates; they are abundant and overlapped at the microscope images.

## 4 Discussion

**Figure 9:**
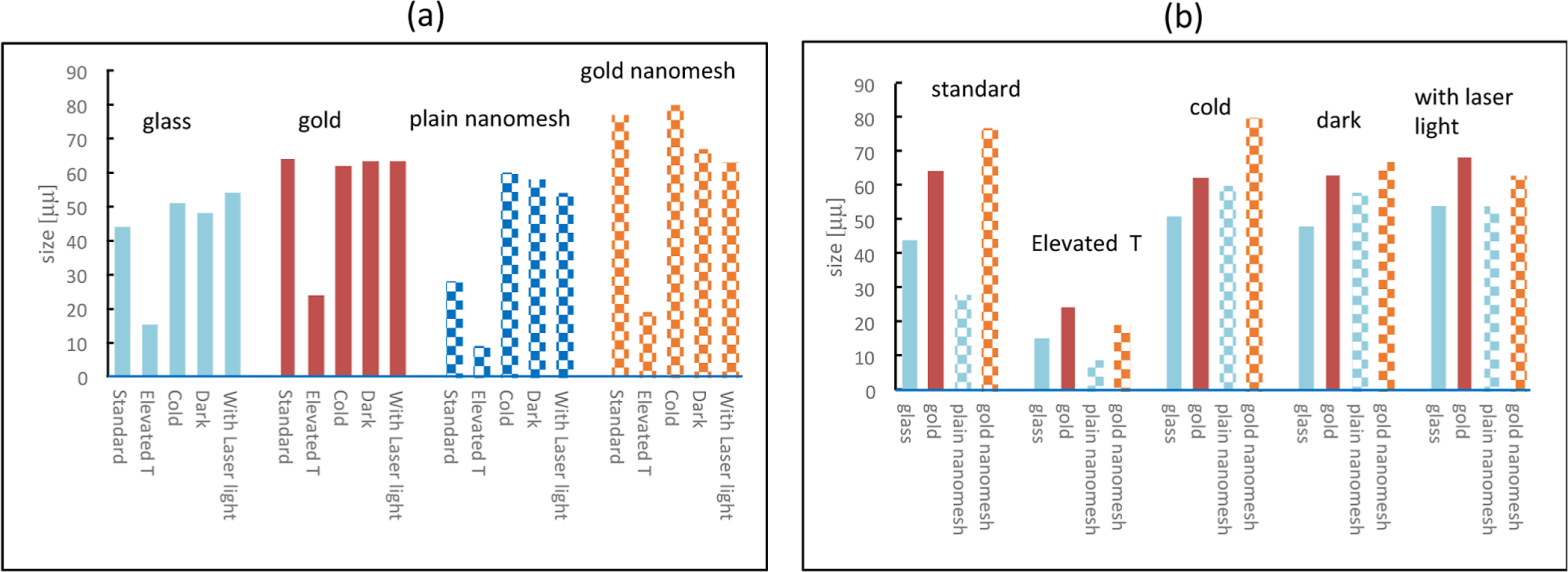
Size of the crystals at (a) different substrates and (b) different environmental conditions.

According to the experiments, microcrystals formed on various substrates and under different experimental conditions strongly differ in shape, size, and substrate coverage. For the estimation of sizes, we measured about 40–60 individual crystals on microscope images, where the crystals can be clearly seen. We also give a rough estimate for a degree of coverage, ranking as low (where crystals are far from each other, as seen, for example, in Figure 6c), intermediate (individual crystals are clearly seen, Figure 6a), and high (crystals cover most of the surface, Figure 8, bottom row). The results are shown in Table 2 and Figure 9.

**Table 2:** Lengths and Substrate Coverage for Er(NO_3_)_3_(Phen)_2_ Microcrystals Grown under Varying Environmental Conditions

	condition	length (μm)	coverage
glass	standard	44	intermediate
	elevated T	15.2	intermediate
	low T	51	high
	dark	48	high
	w/laser light	54	high
flat gold	standard	64	high
	elevated T	24	high
	low T	62	high
	dark	63	high
	w/laser light	63	high
plain nanomesh	standard	28	low
	elevated T	9	low
	low T	60	high
	dark	58	intermediate
	w/laser light	54	intermediate
gold nanomesh	standard	77	intermediate
	elevated T	19	low
	low T	80	high
	dark	67	high
	w/laser light	63	intermediate

In Figure 9a, a table of the results is combined and shown with respect to the substrates, where the crystals are formed and grown. As can be surmised, crystals grow with increased sizes at gold (both plain and nanostructured). However, the coverage (Table 2) is typically higher in plain gold. Figure 9b shows the sizes combined with the dependence on the experimental conditions. The cold and dark conditions are favorable for most cases. The elevated temperatures negatively affect the sizes and shapes of the crystals for all substrates, especially for the plain nanomesh. However, the coverage is still high for plain gold. Thus, it can be concluded that gold stimulates crystal growth. Average sizes of crystals at gold nanomesh tend to be even higher than at plain gold. This is in line with reports of modification of various processes in the vicinity of metal and accelerations of chemical and electrochemical reactions in gold nanostructures.^21−23,25−28^ Gold particles can stimulate the nucleation and crystal growth, as is reported in ref (27) for the crystallization process of lysozymes.

Plasmonic gold can serve as a photocatalytic agent, stimulating the transformation of light to chemical energy and leading to the acceleration of various molecular transformations.^28^ A positive role of the additional illumination on the electrochemical growth of the polyaniline film on the gold substrate is reported in ref (29). In many cases, the role of the nanostructured metal environment is associated with intense light fields and local heating caused by plasmon resonances.^17,25,27^ Excitation of plasmon resonances in nanostructures can also produce hot electrons^26^ or induce electric potentials,^30^ which can affect the processes in their vicinity. However, we believe that this is not the mechanism for the crystal growth enhancement by gold observed in our experiments. Direct illumination does not efficiently excite plasmons in flat gold, and the illumination wavelength does not correspond well to the plasmon dips associated with plasmonic resonances in the nanomesh. In this case, laser light illumination serves as an additional factor on its own, which may or may not stimulate crystal growth. According to our results, additional light does not affect crystal growth. We do not see any significant effect of the laser light illumination on the crystal sizes or coverage, pointing to the fact that local heating associated with light illumination is not the mechanism for the enhanced crystal growth on gold substrates. On the other hand, cold conditions seem to be more favorable for crystal growth. We can speculate that other mechanisms can play a positive role in the vicinity of gold, such as modifications of nanoscale forces and charge transfer processes, which are reported in the vicinity of plasmonic structures and metamaterials^22^ and are ascribed to modifications in the density of photonic modes.^21^ Further studies are needed to clarify the origin of the observed effects.

## 5 Conclusions

In conclusion, using a simple solution growth approach, several types of organic luminescent crystals with Phen as a ligand and Tm, Yb, Gd, La, Nd, Eu, and Er were grown and characterized. The shape and size of the crystals depend on their composition. Choosing the ErPhen system, which grows on thin plates under ambient conditions, we explore microcrystal formation and growth under different experimental conditions and on different substrates. Lower temperatures and dark environments are found to be favorable for crystal growth. The use of gold substrates significantly enhances the crystal growing process.
